# Assessing the Accuracy of Adherence and Sexual Behaviour Data in the MDP301 Vaginal Microbicides Trial Using a Mixed Methods and Triangulation Model

**DOI:** 10.1371/journal.pone.0011632

**Published:** 2010-07-21

**Authors:** Robert Pool, Catherine M. Montgomery, Neetha S. Morar, Oliver Mweemba, Agnes Ssali, Mitzy Gafos, Shelley Lees, Jonathan Stadler, Andrew Nunn, Angela Crook, Richard Hayes, Sheena McCormack

**Affiliations:** 1 Centre for International Health Research (CRESIB), University of Barcelona, Barcelona, Spain; 2 HIV Prevention Research Unit, Medical Research Council, Durban, South Africa; 3 Department of Community Medicine, University of Zambia, Lusaka, Zambia; 4 MRC/UVRI Uganda Research Unit on AIDS, Medical Research Council UK/Uganda Virus Research Institute, Entebbe, Uganda; 5 Africa Centre for Health and Population Studies, University of KwaZulu-Natal, Mtubatuba, South Africa; 6 Infectious Disease Epidemiology Unit, London School of Hygiene and Tropical Medicine, London, United Kingdom; 7 Reproductive Health and HIV Research Unit, Witwatersrand University, Johannesburg, South Africa; 8 Medical Research Council Clinical Trials Unit, London, United Kingdom; 9 Centre for Global Health and Inequality, University of Amsterdam, Amsterdam, The Netherlands; Tulane University, United States of America

## Abstract

**Background:**

Accurate data on adherence and sexual behaviour are crucial in microbicide (and other HIV-related) research. In the absence of a “gold standard” the collection of such data relies largely on participant self-reporting. The Microbicides Development Programme has developed a mixed method/triangulation model for generating more accurate data on adherence and sexual behaviour.

**Methodology/Principal Findings:**

Data were collected from a random subsample of 725 women using structured case record form (CRF) interviews, coital diaries (CD) and in-depth interviews (IDI). Returned used and unused gel applicators were counted and additional data collected through focus group discussions and ethnography. The model is described in detail in a companion paper [1]. When CRF, CD and IDI are compared there is some inconsistency with regard to reporting of sexual behaviour, gel or condom use in more than half. Inaccuracies are least prevalent in the IDI and most prevalent in the CRF, where participants tend to under-report frequency of sex and gel and condom use. Women reported more sex, gel and condom use than their partners. IDI data on adherence match the applicator-return data more closely than the CRF. The main reasons for inaccuracies are participants forgetting, interviewer error, desirability bias, problems with the definition and delineation of key concepts (e.g. “sex act”). Most inaccuracies were unintentional and could be rectified during data collection.

**Conclusions/Significance:**

The CRF – the main source of self-report data on behaviour and adherence in many studies – was the least accurate with regard to measuring sexual behaviour, gel and condom use. This has important implications for the use of structured questionnaires for the collection of data on sexual behaviour and adherence. Integrating in-depth interviews and triangulation into clinical trials could increase the richness and accuracy of behavioural and adherence data.

## Introduction

The accurate measurement of product use and sexual behaviour is extremely important in microbicide and other related clinical trials. First, poor adherence reduces the chance of demonstrating effectiveness. If a trial shows overall benefit then relating the level of protection to adherence is valuable in interpreting the results, and has important implications for predicting effectiveness in real-life settings. Also, in order to properly interpret the results of trials that do not show a protective effect, it is necessary to be able to identify to what extent this may be due to the product not being efficacious, participants not using it or not using it correctly, participants increasing protective behaviours such as condom use, increased risky behaviour related to perceived protection of the product, or other high-risk behaviours [2], [3]. Second, the use of investigational microbicides may negatively affect participants, either directly as a result of harmful side effects or indirectly as a result of changes in behaviour. Having accurate data on product use and related behaviour is important for assessing safety [2], [3]. Third, understanding the reasons for different levels of adherence provides insights that are useful for the design of future clinical trials and for facilitating rollout and access if the product proves effective. Finally, understanding the reasons for non-adherence and for not reporting or inaccurately reporting non-adherence and other relevant behaviours is also important because it can be fed back into the trial and used to improve adherence and the accuracy of adherence data. Similarly, understanding the issues involved in the inaccurate reporting of sexual behaviour and other relevant practices during the trial makes it possible to adjust data collection techniques and improve accuracy.

In the absence of a “gold standard” the collection of data on adherence, and sexual and other sensitive behaviour relies largely on participant self-reporting, the limitations of which are well recognised. In order to overcome these limitations in the Microbicides Development Programme (MDP) – an international partnership set up to evaluate vaginal microbicides to prevent HIV transmission (www.mdp.mrc.ac.uk) – developed a mixed method and triangulation model to collect data on sexual behaviour and adherence in the MDP301 trial. This was a multi-centre, randomised, double-blind, placebo controlled trial that aimed to determine the efficacy and safety of PRO-2000 gel in preventing vaginally acquired HIV infection. It was carried out at six research centres: three in South Africa, and one each in Zambia, Uganda and Tanzania and enrolled 9,385 women who were followed up for 12 months post-randomisation (24 months in Uganda) [4]. The results of the MDP301 trial were announced in late 2009, showing no evidence that PRO-2000 provided protection against HIV infection.

This paper presents some of the data on sexual behaviour and gel use collected using this model, revealing inconsistencies between different methods and inaccuracies located mainly in the structured case record form (CRF) interviews carried out in a clinic setting – the main source of behavioural and adherence data in many clinical trials. In addition, it reveals the nature and location of inaccuracies and some of the misunderstandings arising in the data collection process. It also shows that these inaccuracies are largely unintentional, and that it is possible to identify and correct most of them relatively easily through the use of mixed methods and triangulation during the trial.

## Methods

All trial participants had four-weekly clinic visits during which they received gel supplies and condoms, returned used and remaining unused gel applicators, and were interviewed in the clinic using a case record form (CRF). The visits at weeks 4, 24, 40 and 52 were longer, including clinical examinations and a more detailed CRF interview, containing questions about gel use, vaginal washing and other practices, and detailed questions on each sex act during the last week (or four weeks if the participant did not have sex in the last week). A subsample of 725 women (7.7% of the trial population) was randomly assigned to the social science component of the trial, which was responsible for the triangulation process. The triangulation procedures were linked to three of these long clinic visits, at weeks 4, 24 and 52.

Four weeks prior to these visits the women in the subsample received a coital diary (CD) in which they recorded how many times they had had sex and what kind of sex, whether or not they had used the gel or a condom, vaginal hygiene practices, and various other things. During the clinic visit they handed in their CD and all used and unused gel applicators, which were counted. A member of the clinic staff then interviewed them about the same topics as those in the CD using a structured case record form (CRF).

Shortly after the clinic interview a member of the social science team copied the key information on sexual behaviour, gel and condom use from the CRF and the CD, as well as applicator return data, onto a comparison form, which was integrated into the in-depth interview guide. This enabled the interviewers to see any inconsistencies at a glance. A few days later a social scientist carried out an in-depth interview (IDI), focusing on the same period as the CD and the CRF and on the same behavioural and product-related topics, but in a more open and informal manner. Answers to the questions on sexual behaviour and gel and condom use were also noted on the comparison form. The interviewer also probed to find out the reasons for any discrepancies between the data from different methods, and attempted to establish the most accurate answer in discussion with the participant. The final corrected result was recorded on the comparison form. These interviews were recorded digitally. Consenting male partners of participants who agreed were also interviewed about sexual behaviour during the same period.

The in-depth interview guide also contained a summary section with pre-coded answers and summary fields so that the interviewer could fill in the major findings during or immediately after the interview. These data, together with key data from the CD and the comparison form, were entered into a summary database that provided quick access to the results in a quantitative format. Where relevant, the information from the above process was fed back to the local clinic teams and to the central Trial Management Group during monthly calls to review progress.

Focus group discussions with trial participants and community members about the gel, the trial, sexual behaviour and related issues were carried out to collect more general information on community attitudes. Ethnography was carried out in the research communities and clinics. Sometimes these activities were aimed at specific problems that arose during the trial.

Transcriptions from the recorded in-depth interviews, FGDs and notes from the ethnography were entered and coded in Nvivo (a software programme for the management and analysis of qualitative data). Continuous analysis of the data was carried out on a site level at the different research centres as well as centrally across all sites.

The mixed method model is described in more detail in a companion paper [1].

Altogether there are 1866 in-depth interviews, most with matching CD, CRF and applicator count data, from the 725 women in the sub-sample. In addition there are 462 interviews with 244 male partners, 100 FGDs with trial participants who were not randomised to this sub sample, 119 FGDs with community members, and extensive ethnographic notes. All the qualitative data have been transcribed and coded in Nvivo (a software package for managing and coding qualitative data).

The discussion in this paper is based on an analysis of in-depth interview, coital diary and CRF data from 1636 clinic visits by 704 women: it only includes visits for which we have complete data on reporting of sex, gel use and condom use from case record forms, coital diaries and in-depth interviews. The focus then narrows to a smaller sample of 1443 visits for which we also have matching applicator return data. The women in these two smaller sub-samples do not appear to be different in any way to those for whom we do not have the full data from all clinic visits. In what follows we shall refer to these collections of data derived from different instruments but collected in relation to the week preceding particular clinic visits as “datasets”.

## Results

### Inconsistency and inaccuracy

Before proceeding we need to define what we mean by consistent and inconsistent, and to clarify the relationship between consistency and accuracy. If a participant gives the same answer to the same question across the three different methods, then we call this consistent (leaving aside the not unimportant philosophical question of the extent to which a question can be considered “the same” in the context of a structured CRF questionnaire, a coital diary and an in-depth interview [1]. If the participant gives different answers, then there is inconsistency. So for example, if a woman reports three sex acts in the CRF interview but reports five sex acts in the in-depth interview and records five in her coital diary, then we consider her reporting of sex to be inconsistent.

This is made easier by the numerical nature of the answers, but complicated by the fact that the behaviours being quantified are not easy to delineate. For example, even though the trial defined “sex act” as “a single act of vaginal penetration, with or without ejaculation,” there is still much ambiguity regarding what counts as a sex act, and the overlap in meaning with local concepts of “sex,” “days” (on which people have sex), and “rounds” (there may be numerous “rounds” of sex in a “day”) is only partial. As a result, the ostensibly unambiguous numbers hide a more ambiguous reality.

**Figure 1:**
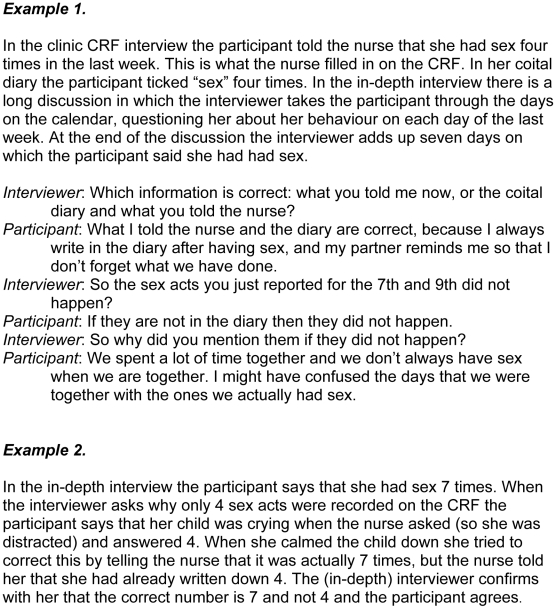
Resolving inconsistency.

If a woman reports the frequency of a particular behaviour consistently across all methods and there is nothing to suggest otherwise, then we take this as the final, triangulated result. If there are inconsistencies in reporting between the methods but these inconsistencies are clarified in a plausible manner during the discussion of inaccuracies in the in-depth interview, then the figure agreed on in that discussion is taken as the final triangulated figure (Figure 1).

We assume that the triangulated figure is the most accurate one on pragmatic grounds, because it is plausible and there is no evidence to the contrary, though of course there is no absolute way of knowing this (here again we are avoiding philosophical discussion). In most of the datasets with some inconsistency it is relatively straightforward to resolve this through probing and discussion with the participant (Figure 1).

### Extent of inconsistencies

**Figure 2:**
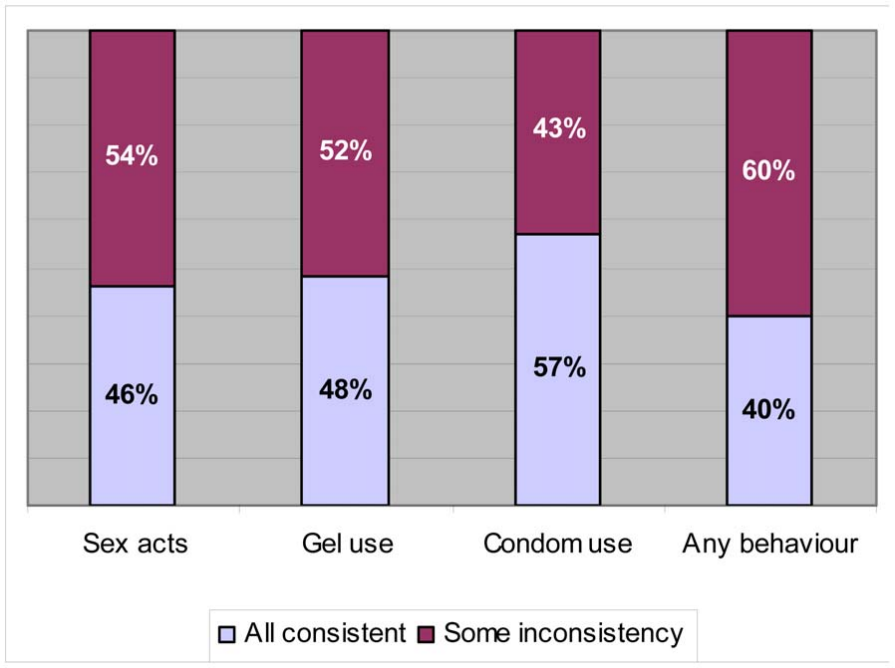
Proportion of datasets in which reporting of number of sex acts, gel use and condom in the last week/4 weeks are consistent/inconsistent.

The triangulation process revealed inconsistencies between the data collected using CRF questionnaires, coital diaries and in-depth interviews. Looking at the reporting of numbers of sex acts in the last week/4 weeks, for example, there were inconsistencies in 54% (876/1636) of the datasets. For the reporting of gel and condom use this was 52% (850/1636) and 43% (705/1636) respectively. Looking at all behaviours together, there was some inconsistency in 60% (983/1636) of the datasets (Figure 2).

Of the 983 datasets with some inconsistency in the reporting of any behaviour (i.e. the 60% in the right-hand column in Figure 2), most involved inaccuracy in the reporting of all behaviours (59%) or the reporting of both numbers of sex acts and gel use (20%). Only 12% of the datasets contained inaccuracies relating to a single behaviour.

Of course, the extent of inconsistency depends on how it is defined. In Figure 2 “some inconsistency” refers to *any* inconsistency, ranging from very large discrepancies in different behaviours and across different methods to small discrepancies in a single method and relating to a single behaviour. In this “pessimistic” scenario, a woman who forgot to report a single sex act in her coital diary would fall into the “inconsistent” category, together with a woman who grossly misreported the number of sex acts, gel use and condom use across different methods. A looser definition or different criteria would result in less inconsistency. For example, if we only compare the reported number of sex acts in the CRF with the triangulated data ± one sex act, then there is a 75% match (i.e. higher than the 63% using perfect agreement), which increases to 82%± two sex acts.

### Differences in accuracy between the methods

Inaccuracies are not evenly distributed between the different methods: if we assume that the triangulated data are most accurate then the inaccuracies in the reporting of numbers of sex acts, gel use and condom use are most prevalent in the CRF and least prevalent in the in-depth interviews. For example, in 63% of the CRF interviews the number of sex acts matches the triangulated figure; for the CD this is 72% and for the IDI 84%. The figures are very similar for condom and gel use (Table 1). Of course, it is more likely that the IDI result will be closer to the triangulated result because the IDI is central in establishing the triangulated result, but there are reasons for arguing that this is more accurate. For example the close match between IDI and CD data, the possibility of probing answers, and the plausibility of answers given in the IDI, and the fact that the probing in the IDI also reveals inaccuracies in the IDI itself.

**Table 1:** Reporting of sex, gel use and condom use across the different methods.

	METHOD	CLINIC VISITS/DATASETS [behaviours]				NO. OF BEHAVIOURS	
		Total	Under-report	Exact match	Over-report	Total (mean)	Diff. with Triangulated
Sex	CRF	1636	496 (30%) [2205]	1025 (63%)	115 (7%) [298]	7362 (4.5)	−1907 (21%)
	CD		317 (19%) [1202]	1183 (72%)	136 (8%) [629]	8696 (5.3)	−593 (6%)
	IDI		147 (9%) [448]	1376 (84%)	113 (7%) [240]	9061 (5.5)	−208 (2%)
	**Triangulated**					**9269 (5.7)**	
Condom	CRF	1636	335 (20%) [1403]	1150 (70%)	151 (9%) [408]	5109 (3.1)	−995 (16%)
	CD		239 (15%) [832]	1258 (77%)	139 (8%) [564]	5838 (3.7)	−266 (4%)
	IDI		97 (6%) [312]	1444 (88%)	95 (6%) [189]	5981 (3.7)	−123 (2%)
	**Triangulated**					**6104 (3.7)**	
Gel	CRF	1636	425 (26%) [1843]	1033 (63%)	178 (11%) [459]	6556 (4.0)	−1389 (17%)
	CD		283 (17%) [1024]	1205 (74%)	148 (9%) [599]	7521 (4.6)	−424 (5%)
	IDI		125 (8%) [385]	1394 (85%)	117 (7%) [260]	7820 (4.8)	−125 (2%)
	**Triangulated**					**7945 (4.9)**	

If we assume that the triangulated figure is the most accurate, then most of the inaccuracy is in the CRF. However, this does not mean that the triangulated result always agrees with the IDI. If, for example, we look only at the 876 datasets that contain some inconsistency in the reporting of number of sex acts, then it is clear that there is substantial inaccuracy in the other methods as well: 68% of the CRFs, 53% of the coital diaries and 30% of the in-depth interviews were inaccurate when compared to the triangulated data.

### Differences in the reporting of the frequency of sex, condom use and gel use between methods

If we compare individual methods, there was more reporting of all behaviours (sex acts, gel use, and condom use) in the coital diary compared to the CRF, and more in the in-depth interview compared to the coital diary. The triangulated numbers are only marginally higher than those from the IDIs. The differences between the coital diaries, the in-depth interviews and the triangulated data are relatively small compared to the difference between these and the clinic CRF: participants reported 21% fewer sex acts, 16% fewer condoms and 17% less gel use in the CRF compared to the triangulated data; this is 6%, 4% and 5% respectively for the CD when compared to the triangulated data (Table 1).

For the last week/4 weeks participants reported a mean number of 4.5 sex acts in the CRF, 5.3 in the CD, 5.5 in the IDI and 5.7 in the triangulated data. The mean number of gels they reported using increases similarly from 4.0 in the CRF to 4.9 in the triangulated data. Reported condom use increases from 3.1 in the CRF to 3.7 in the triangulated data (Table 1).

On an individual level there was both over- and underreporting of behaviours across all the methods. For example, in 30% of the CRF interviews participants under-reported the number of sex acts by 2205 compared to the triangulated data, and in 7% of the CRF interviews they over-reported the number of sex acts by 298 compared to the triangulated data (Table 1).

### Differences between partners

There were also inconsistencies between the participants' reporting and that of their male partners. Altogether there were 462 interviews with 244 male partners of participant women. These interviews covered the same one week/4 week period covered by the women's interviews. However, because of the difficulty accessing the men (due to their work, mobility or reluctance) these interviews were often too long after the women's interview to make them a reliable source of comparison, as the men would be likely to have forgotten the details of their behaviour and the exact days referred to in their partner's interview. As a result, what we present below is based only on the 372 interviews with 195 men that were conducted within one week of their female partner's interview.

On average women reported 10% more sex than their male partners. They also reported 8% more gel use and 16% more condom use. When individual couples are compared, men reported differently to their partner's triangulated number of sex acts in 64% (238/372) of the interviews. In 109 cases the men reported more sex than the triangulated figure for their partners, and in 129 cases they report less (in eight cases she is talking about the last 4 weeks while he means the last week). When questioned about the discrepancy, men tended to say that they thought that their partners' answers were probably more accurate because they were the ones keeping track due to the trial, and because counting how often you had sex was a “woman thing”.

### Comparing self-reported gel use to returned used applicators

The triangulation process based on discussion of inconsistencies with participants and the degree of agreement between the IDI and the CD strongly suggest that the CRF is the least accurate and the IDI the most accurate of the three methods. But we do not know for certain that this is the case, because we do not have any independent, objective source of information on the behaviours in question: we have only compared and triangulated different sets of self-reported data and assessed participants' explanations of the discrepancies. However, we do have one additional source of data on gel use: the gel applicator returns.

During their monthly clinic visits women were required to return all used and unused applicators that they had received at their previous visit. These were counted and recorded on a gel accountability CRF, and women were asked to explain any missing applicators. They then received a new supply of gel, the number of applicators being based on their estimated need for the coming month. For 1443 of the 1636 datasets used for this paper, we also have matching data on returned applicators.

However, triangulating the self-report data on gel use with the gel accountability data is not straightforward. Applicators were collected monthly whereas the behavioural CRF and in-depth interviews focused on the last week, except when women had not had sex in that week, in which case the interviews focused on the last four weeks. As a result, it is only possible to match reported gel use and numbers of used applicators for the same time period for this latter group of women (195/1443 datasets). For the majority of women for whom we have applicator-return data (1248 datasets) it is only possible to estimate average weekly gel use by dividing the number of returned applicators by the number of weeks since the last clinic visit. This makes anything more than an approximate match for these women unreliable.

Bearing these limitations in mind, a comparison of self-reported gel use from the different methods reveals that the total number of returned used applicators matches the CD, IDI and triangulated data more closely than the CRF. If we assume for the moment that the number of used applicators represents the number of gels actually used as intended, then overall there is some over-reporting of gel use in the IDIs and triangulated data and under-reporting in the CD and CRF, with the IDI figures before triangulation being closest to the applicator returns (Table 2).

**Table 2:** Total number of reported gels used by method compared to the number of returned used applicators.

Method	Number of gels used
CRF	5897
CD	6908
IDI	7051
Triangulated	7165
Used applicator count	7001

As with the figures for numbers of sex acts, the cumulative figures for reported gel use conceal individual variations in reporting.

### Reasons for discrepancy between data from different sources

From the discussion in the IDIs, many reasons for the differences in reporting of sexual behaviour and gel use between the different methods became clear. They tend to fall into four main categories. These categories are not exclusive, however, and some of the issues discussed below fit under more than one heading.

#### Practical

1. Participants forgetting (particularly during the CRF interview) how often they had had sex and how many gels and condoms they had used.
2. CRF interviewers not probing when the answer was unclear or contradictory, misunderstanding answers, not listening properly to answers, writing answers down incorrectly (Figure 1, example 2), and even not asking the question but filling in an answer anyway.

#### Desirability

**Figure 3:**
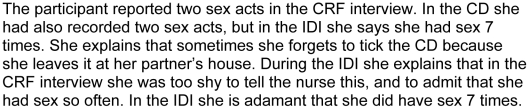
Desirability bias.

3. Desirability bias involves participants giving the answer that they think the interviewer wants to hear, or not giving the answer they think the interviewer might disapprove of. Desirability bias led to under-reporting of sex (and consequently also gel and condom use) in the CRF because some participants perceived themselves as having a lot of sex and were embarrassed to report this to clinic staff. These women did loosen up and discuss this in the context of the more informal in-depth interviews (Figure 3).

#### Definition and delineation

**Figure 4:**
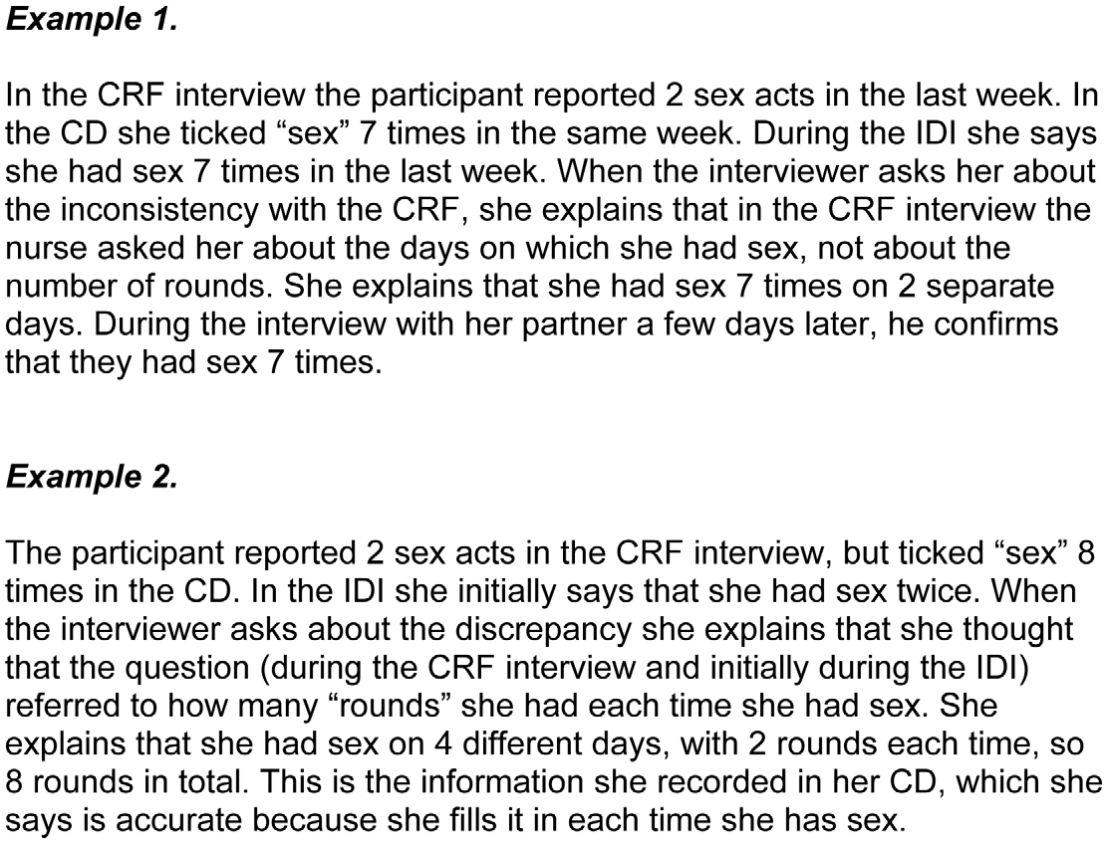
Category misunderstandings.

4. Much of the under- and over-reporting stemmed from the way in which various categories were defined and delineated by both participants and researchers. The most common misunderstandings related to the definition of “sex act” (Figure 4).
5. Sometimes participants who had had sex in the last week were nonetheless recorded as only having had sex in the last four weeks during the CRF interview. As a result one week's self-reported gel use was compared to a month's returned applicators (see the section “Comparing self-reported gel use to returned used applicators” above).
6. In the case of participants who had not had sex in the last week and were supposed to be questioned about the last four weeks, CRF interviewers sometimes asked participants about “this month” rather than “the last four weeks,” thus missing the sex acts and gel use in the earlier weeks if the interview fell in the middle of the month.
7. Participants sometimes used gel without having sex, and such use was usually not reported in the CRF interview or the CD as “gel use” (but did come up in the IDI). For example, some wanted to try out the applicator or demonstrate to their partner. Sometimes participants used gel to “cleanse” their vagina, and there were unsubstantiated rumours (in the FGDs and community ethnography) of some women using the gel as hair gel or skin cream. As a result, used applicators did not necessarily mean the gel had been used for sex.
8. Although they were not questioned explicitly about this, it seems likely, from some of the IDI discussions in which participants reported having five or six “rounds” during the course of a single evening, that they did not insert a new gel for each round but did assume when they were being questioned that all the rounds were “sex acts with gel”. In this case, conversely to point 7 above, lack of used applicators did not necessarily mean that gel was not used during sex.

#### Deception

9. It is possible that some participants shared gel with other women or squeezed out gel in order to return empty applicators to the clinic. Although there is some evidence of limited gel sharing, with a few participants admitting to it (for example a women running out of gel and borrowing some from a friend), there is only very limited indirect evidence – from the focus group discussions and ethnography – of participants deliberately dumping gel. Also, because the applicators were dispensed in boxes of ten and used applicators returned in the same boxes, some women may have emptied the last few applicators in order to return full boxes. Although a few participants said that they knew of other women squeezing out the gel to empty the applicators, none ever admitted doing this themselves.

## Discussion

In this paper we have shown how a mixed method and triangulation model, described in detail in a separate paper [1], was used to reveal inconsistencies in the data on sexual behaviour, gel use and condom use collected using closed structured interviews, coital diaries and in-depth interviews. The paper has also described the attempt to resolve these inconsistencies through dialogue with study participants and triangulation. We have also compared self-reported gel use with the number of used gel applicators that participants returned to the clinic and discussed some of the problems inherent in this comparison. We conclude that data from the clinical CRF – the main source of self-report data on behaviour and adherence in many studies – was least accurate with regard to measuring sexual behaviour and gel use. This has important implications for the use of structured questionnaires for the collection of data on sexual behaviour and adherence in microbicide and other similar clinical trials, and suggests that integrating in-depth interviews and triangulation into trials could increase both the accuracy and the richness of behavioural and adherence data, as well as contributing to an understanding of the reasons for inaccurate reporting. The in-depth interview is the key to the triangulation process because it enables critical discussion with the participant and, after it has been transcribed and coded, it can be independently assessed and subjected to deeper interpretation.

Having said that, we need to consider the limitations. We have assumed that the triangulated result is the most accurate one, based on consistency of the data from different sources, verification by the participant, consistency within the IDI text when it is analysed in detail, numbers of returned gel applicators, and the absence of evidence to the contrary. This assumption is plausible, and our conclusions are supported by the figures for used applicator returns. But it is not possible to be completely certain.

Because the triangulation/resolution process is an intrinsic part of the IDI, and is conducted by the same interviewer, it could be argued that the triangulated result is dependent on the skills of that interviewer. This argument is correct, and the extent to which inconsistencies are revealed and solved is clearly related to the skill of the interviewer. And many of the problems that have emerged from the CRF interview (and the mistakes made in the IDI) are clearly related to the lack of interviewer skills. This serves to emphasise the importance of recruiting those with the appropriate interpersonal skills and investing in adequate training to go beyond the simple reading of questions from a questionnaire and superficial probing.

It could also be argued that there may be a bias towards “accepting” the accuracy of the (initial) IDI result, since respondents may find it easier to say they have given misleading information to a clinic interviewer earlier in the process than to admit, face-to-face, that they have just misled the IDI interviewer. However, the triangulation process reveals that 30% of the IDIs themselves contain inaccuracies, and in these cases it has been concluded that the CD or the CRF was more accurate, quite often as a result of the participant herself insisting on this (see Figure 1, example 1 and Figure 4, example 2 for participants claiming that the CD was correct).

The process not only generated richer and plausibly more accurate data, but also revealed weaknesses and errors across the whole data-collection process: the mistakes of the interviewers, the shortcomings of the closed and structured interview process, the errors in the data, the difficulties inherent in the categories and concepts used. Some of these could have been avoided by better training, more monitoring, and better quality control; others were new and unexpected. So the process has been a double-edged sword: more detail and more accurate data at the price of revealing our mistakes and weaknesses. But this has also made it a good learning experience for future studies.

And the increased “accuracy” still remains contentious to some extent – the product of comparing results from different but imperfect methods and interpretation, rather than the “gold standard” craved by clinical trialists. However – and this is another positive aspect of the learning process – we also realise that a validated biomarker for sex or gel use would not solve all the issues and problems identified here: we would still need to ensure that mistakes were not made in defining and recording time periods, that researchers and participants were using terms and concepts in the same way, that relevant behaviours for which there is no biomarker were accurately reported, etc. A validated biomarker would improve the overall accuracy and the comprehensiveness of the process (as would better coital diaries, better interviews, and better quality control), rather than being an alternative. Triangulation should therefore be seen broadly, as contributing to a more accurate *and* a more comprehensive picture, rather than being narrowly focused on validating the accuracy of a single measure.

We have revealed substantial inconsistency between the data from different methods, but the extent of these inconsistencies is also partly a result of the narrow definition of inconsistency: a looser (and perhaps more realistic) definition results in fewer inconsistencies (just as a looser definition of adherence results in higher adherence). This implies that some critical discussion is needed about how much inaccuracy is acceptable and what constitutes sufficient adherence.

On the one hand, the results presented above could be interpreted as revealing a worrying level of inconsistency in what is generally supposed to be accurate data. Yet, given the complexity of the behaviours being measured, the fallibility of human memory, and the extent of things that can go wrong in measuring such behaviour through self-report, it is perhaps surprising how well the reported number of sex acts in the CRF matched the triangulated figure, and how closely the reporting of gel use in both the CRF and the triangulated data matched the number of returned used applicators.

The data suggest that much of the inaccurate reporting is unintentional and relatively easy to remedy through simply discussing inconsistencies with participants. In the early stages of designing the methodology there was some resistance to “confronting” participants with discrepancies in their reporting. However, it is clear that if this is done sensitively then participants do not find it threatening, and indeed welcome the opportunity to correct unintentional errors that they themselves are not happy about. Importantly, the data show that we should not assume that study participants are generally prone to *intentional* deception.

It is also clear that, in spite of the generally high levels of staff competence, training, quality control, etc. that characterised the MDP301 trial, errors still occurred, and integrated social science studies such as the one described in this paper and the companion paper make it possible to identify these and suggest further measures to avoid them (for example, in this case through trying to improve participants' recall by developing better memory aids or simplifying questioning (asking only about the last act rather than the last week), or reducing desirability bias by designing better participant information procedures. There are examples of other innovative approaches to some of these issues in the literature, for example the use of interactive computer-based participant education for informed consent [5], and the use of educational video to improve HAART adherence [6].

The data also suggest that interviews with male partners do not add much to the triangulation process in this type of study. Male partners were difficult to recruit and follow up: they tended to be mobile, employed, and generally reluctant to participate. It was also difficult to probe about inconsistencies in the participant's reporting of sex because these may have been related to multiple partners, and we were aware of the ethical and social and implications raising such issues too critically during interviews with male partners. Triangulating interviews with male partners may be more meaningful where men and women are recruited together as a couple; in a safety and acceptability study of Carraguard vaginal gel in Thai couples, for example, it was found that sex and gel use were well correlated in partners' reports [7].

Finally, it might seem counter-intuitive that participants tended to *under*-report gel and condom use in the CRF. Given that they were repeatedly told by clinic staff that they should use gel and a condom whenever they had sex, it might be expected that they would be more inclined to over-report when asked about this in the clinic. This was probably due to the fact that gel and condom data were collected per sex act and, because some women under-reported the number of sex acts due to embarrassment about reporting what they considered to be too much sex to clinic staff, they also had to under-report gel and condom use.

### Conclusions

This paper has described some of the results relating to the accuracy of sexual behaviour and adherence data obtained by integrating qualitative and anthropological methods into a large multi-site clinical trial and triangulating the results. The evidence from this process suggests that there are significant inaccuracies in the behavioural and adherence data collected using structured CRF interviews in a clinic setting – the main source of such data in many Phase III HIV prevention trials. However, the data also show that these inaccuracies are largely unintentional, and that it is possible to identify them relatively easily through triangulation and correct most of them through the integration of dialogue with participants.
